# Comments: "Association between lipoprotein(a) and coronary heart disease risk in type 2 diabetes mellitus and evaluation of statin treatment effects"

**DOI:** 10.1590/1806-9282.20250675

**Published:** 2025-10-17

**Authors:** Sami A. Gabr

**Affiliations:** 1 Mansoura University, Faculty of Medicine, Department of Medical Microbiology and Immunology, Genetics of Microbiology Unit, Biochemistry and Molecular Biology – Mansoura, Egypt.

## COMMENTARY

Heart disease remains a major cause of morbidity and mortality among individuals with type 2 diabetes. A pooled relative risk associated with biomarkers, including elevated lipids, were presented for the incidence of coronary heart disease (CHD) in adults with diabetes^1,2^.

Classic heart disease risk markers, including elevated low-density lipoprotein-cholesterol (LDL-C), elevated blood pressure, smoking, elevated triglycerides, and low high-density lipoprotein-cholesterol, have been clearly demonstrated to be important determinants of heart disease in individuals with diabetes. However, the association between lipoprotein(a) [Lp(a)] and CHD risk in individuals with type 2 diabetes mellitus (T2DM) is an area of growing research interest, particularly regarding the impact of statin therapy. Recent studies have reported that Lp(a) might be an independent risk factor for CHD in T2DM patients. Thus, lipid-lowering therapy with statins alone cannot reduce Lp(a) levels in diabetic patients with CHD risk^1-3^.

Lp(a) is a unique lipoprotein particle that consists of low-density lipoprotein (LDL) molecules bound to a specific glycoprotein called apolipoprotein(a). Lp(a) levels are largely determined genetically and are less influenced by lifestyle or dietary modifications. Elevated Lp(a) concentrations are recognized as an independent risk factor for cardiovascular diseases, including CHD^3-5^.

In the context of T2DM, the role of Lp(a) in the pathogenesis of CHD is of particular concern. T2DM itself is a well-established risk factor for atherosclerotic cardiovascular diseases, but the contribution of Lp(a) in this population remains complex and multifactorial. Several pathophysiological mechanisms have been proposed to explain the association between Lp(a) and the prognosis of CHD in T2DM, including the atherogenicity of Lp(a), its pro-inflammatory and pro-thrombotic effects, and diabetic dyslipidemia^6-9^.

Lp(a) may further complicate the dyslipidemic profile in diabetic patients, as its pro-atherogenic and pro-inflammatory effects combine with other lipid abnormalities to enhance the risk of CHD. The role of elevated Lp(a) in predicting cardiovascular risk, particularly in patients with type 2 diabetes, has shown to be associated with an increased risk of major adverse cardiovascular events (MACEs) and coronary artery disease (CAD) in patients with longer durations of T2DM, independently of other known risk factors such as LDL-C and HbA1c levels^8-11^.

Finally, the relationship between Lp(a) and CHD in T2DM patients underscores the complexity of cardiovascular risk assessment in this population. While statins remain foundational in reducing the overall cardiovascular disease risk, they are insufficient to fully address the risks posed by elevated Lp(a) in some individuals^3^. Thus, clinical trials are ongoing, and several novel therapeutic approaches, including PCSK9 inhibitors and antisense oligonucleotides, that offer hope for more effective management of elevated Lp(a) levels have been explored^12-15^.

## Data Availability

The datasets generated and/or analyzed during the current study are available from the corresponding author upon reasonable request.
